# Aujeszky’s Disease in a Grey Wolf (*Canis lupus*) in Poland

**DOI:** 10.3390/v18040449

**Published:** 2026-04-08

**Authors:** Wojciech Wójcik, Anna Didkowska, Blanka Orłowska, Sabina Nowak, Bartosz Sell, Krzysztof Anusz, Florian Pfaff, Bernd Hoffmann

**Affiliations:** 1 “Dziki Projekt” Foundation, Tuwima 8 St., 38-300 Gorlice, Poland; fundacjadzikiprojekt@gmail.com; 2 Department of Food Hygiene and Public Health Protection, Institute of Veterinary Medicine, Warsaw University of Life Sciences (SGGW), Nowoursynowska 159 St., 02-787 Warsaw, Poland; anna_didkowska@sggw.edu.pl (A.D.); blanka_orlowska@sggw.edu.pl (B.O.); krzysztof_anusz@sggw.edu.pl (K.A.); 3 Department of Animal Ecology and Evolution, Institute of Ecology, Faculty of Biology, University of Warsaw, Żwirki i Wigury 101, 02-089 Warszawa, Poland; sabina.pieruzeknowak@gmail.com; 4 Department of Chemical Research of Food and Feed, National Veterinary Research Institute, Partyzantow 57, 24-100 Puławy, Poland; bartosz.sell@piwet.pulawy.pl; 5 Friedrich-Loeffler-Institut, Federal Research Institute for Animal Health, Südufer 10, 17493 Greifswald-Insel Riems, Germany; bernd.hoffmann@fli.de

**Keywords:** wolves, Aujeszky’s disease, pseudorabies, suid herpesvirus 1, genomics

## Abstract

Aujeszky’s disease (AD), caused by suid herpesvirus 1 (pseudorabies virus, PRV), is a highly contagious infection primarily affecting swine, with wild boars serving as an important reservoir in Europe. Spillover infections in non-suid species, including carnivores, are rare but typically fatal and of epidemiological concern. This study presents the first case of AD in a grey wolf (*Canis lupus*) in Central Europe with genomic characterization. The 8-month-old wolf was found in the Carpathians (SE Poland), moribund with acute neurological signs, and euthanized for animal welfare reasons. Necropsy revealed no pathognomonic gross lesions. Molecular analyses of tissues confirmed the presence of PRV DNA using real-time PCR, and virus isolation was successful. Genomic analysis revealed that the PRV isolate clustered within genotype I, the predominant circulating genotype in Europe. However, due to the limited availability of reference PRV genome sequences from European wildlife, the precise geographic origin and transmission pathways of this strain could not be fully resolved. In the presented case, wild boars were considered a possible source of infection. This highlights the potential for PRV transmission to apex predators. This study emphasizes the importance of systematic surveillance of PRV in wildlife and the need for expanded genomic databases of PRV strains. Full-genome sequencing is crucial for improving the understanding of PRV transmission.

## 1. Introduction

Pseudorabies virus (PRV), also known as suid alphaherpesvirus 1, belongs to the species *Varicellovirus suidalpha1* within the family *Orthoherpesviridae*, subfamily *Alphaherpesvirinae*, genus *Varicellovirus* [1,2]. PRV is the causative agent of Aujeszky’s disease (AD) in pigs. Due to its wide host range, PRV can infect many mammalian species, and these infections almost always result in death as a consequence of the virus’s strong neurotropism and the severe inflammatory response it induces [3,4]. Pigs are the only species that can survive a productive PRV infection, and consequently, pigs are regarded as the natural hosts of PRV. In contrast, higher primates, including humans, as well as equids, are resistant to infection [3].

AD is classified as a C-listed disease in the European Union, according to the Commission Implementing Regulation (EU) 2018/1882 of 3 December 2018. Vaccination programs and culling have eradicated AD from domestic swine (*Sus scrofa domesticus*) in many European countries [5,6], including Poland (AD-free status since 2023) [7]. The main host of AD remains wild boar (*S. scrofa*), due to its worldwide distribution and good adaptation potential [8,9]. In Poland, data from 2024 showed a PRV seroprevalence of 32.19% in wild boars [10], which is consistent with earlier studies [11]. Even though PRV spillover infections are most often observed in farm dogs, recently, more and more cases have been noted in hunting dogs in Europe [12,13,14,15]. Lastly, cases of PRV infections are being reported in wildlife carnivores, among others, in red fox (*Vulpes vulpes*) [16,17], Iberian lynx (*Lynx pardinus*) [18], and grey wolf (*Canis lupus*) [19,20,21,22].

In Poland, the wolf population, fully protected since 1998 [23], has recently expanded nationwide [24,25]. The main prey of Polish wolves are wild ungulates, including roe deer (*Capreolus capreolus*), red deer (*Cervus elaphus*), and wild boars, which account for 45–97% of their food biomass, with medium-sized mammals, mainly Eurasian beaver (*Castor fiber*), supplementing their diet [26,27,28].

This paper describes the first case of AD in a wolf in Poland and the genetic characterization of the isolated strain.

## 2. Materials and Methods

### 2.1. Field Investigation and Samples

**Figure 1:**
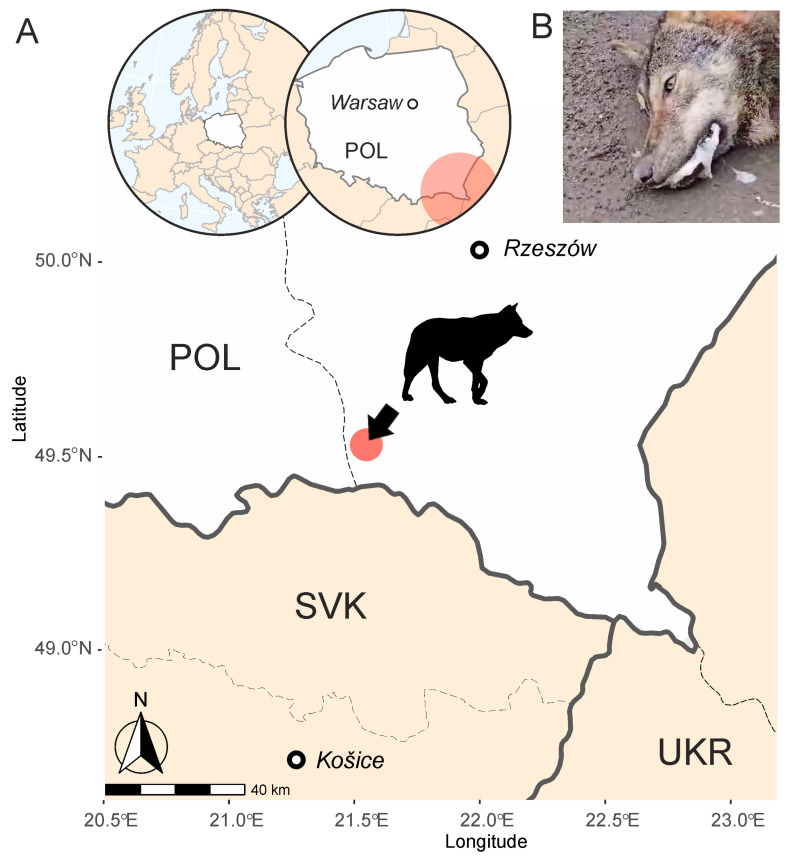
(**A**) Map indicating the location in south-east Poland (Magura National Park), where the wolf was found. (**B**) The wolf was found moribund showing severe epileptic seizures and excessive salivation as seen in this screenshot from Video S1.

In February 2025, a ten-month-old male wolf was found near death and exhibiting severe neurological symptoms in Magura National Park, in the Carpathian Mountains (south-eastern Poland, Figure 1). An autopsy was subsequently performed. During the procedure, tissue samples were collected from the heart, liver, spleen, lungs, kidneys, intestine, mesenteric lymph nodes, thymus, and bladder and then frozen for subsequent analysis. During the necropsy, a radiographic examination was performed to exclude the presence of metallic foreign bodies (bullets or bullet fragments).

### 2.2. Toxicological Screening

For the toxicological examination of samples, we use a previously described and validated method [29]. Detailed information regarding the calibration ranges, recovery efficiencies, and limits of quantification for the analyzed compounds is provided in Sell et al. 2018 [29].

Before analysis, the liver and stomach content samples were thawed and homogenized. A 2 g aliquot of each sample was mixed with 5 mL of acetonitrile and vortexed for 30 s. Subsequently, 0.5 g of sodium acetate was added, followed by a 1 min vortex. The mixture was sonicated for 15 min in an ultrasonic bath, vortexed again for 1 min, and centrifuged at 2930× *g* for 10 min. A 0.7 mL portion of the supernatant was transferred to a clean centrifuge tube containing 150 mg of MgSO_4_, 50 mg of C18, and 50 mg of primary–secondary amine (PSA). After vortexing for 1 min and centrifuging at 2930× *g* for 10 min, the supernatant was filtered through a 0.2 μm membrane and transferred to autosampler vials.

Toxicological screening was performed using a validated multi-residue method based on liquid chromatography coupled with tandem mass spectrometry (LC-MS/MS). The analysis was conducted using a Shimadzu LC system (Shimadzu, Kyoto, Japan) connected to an ABSciex API 5500 Qtrap mass spectrometer (AB SCIEX, Concord, ON, Canada).

Chromatographic separation was achieved on a C8 column (75 mm × 2.1 mm, 3 μm particle size) using a gradient elution with a mobile phase consisting of 5% isopropanol in ethanol and 0.5% isopropanol in 0.1% acetic acid in water. The mass spectrometer operated in negative and positive ionization modes, and transitions for potentially toxic substances were monitored in multiple reaction monitoring (MRM) mode.

### 2.3. Rapid Lateral Flow Immunochromatographic Tests

Rapid lateral flow immunochromatographic tests were performed to detect the presence of antigens for canine parvovirus (CPV), canine distemper virus (CDV), canine coronavirus (CCV), canine adenovirus (CAV), canine influenza virus (CIV), *Giardia*, *Dirofilaria immitis*, and antibodies against *Ehrlichia canis*, *Borrelia burgdorferi*, *Anaplasma phagocytophilum*, and *A. platys*. The tests were carried out according to the manufacturer’s instructions using Vet Expert Rapid Tests (Vet Planet, Warsaw, Poland).

### 2.4. Direct Immunofluorescence Assay

The presence of lyssaviruses was tested using the reference method, direct immunofluorescence assay (DFA), in accordance with the Polish Chief Veterinary Officer Instruction (No. GIWpr-02010-2/2018, [30]). Briefly, DFA was performed on brain impression smears using a monovalent anti-nucleocapsid conjugate. Fresh brain tissue impressions were prepared on glass slides, air-dried, and fixed in cold acetone. The slides were then incubated with a fluorescein isothiocyanate (FITC)-labeled monovalent anti-nucleocapsid antibody. After washing, the slides were examined under a fluorescence microscope. The presence of specific apple-green fluorescence within neuronal cells was considered a positive result.

### 2.5. Nucleic Acid Extraction and Real-Time PCR

Organ samples were homogenized in 800 µL serum-free medium with antibiotics using the TissueLyser II tissue homogenizer (Qiagen, Hilden, Germany). Genome extraction of homogenized tissue samples and cell culture material was performed utilizing the KingFisher Flex System (Thermo Scientific, Darmstadt, Germany) using the NucleoMag Vet kit (Macherey-Nagel, Düren, Germany) according to the manufacturer’s instructions.

The extracted nucleic acid was amplified using the published real-time PCR assays for PRV [31], canine distemper virus [32], influenza A virus [33], and canine herpesvirus 1 [34]. In all assays, an internal process control based on beta-actin was co-amplified [35]. All RT-qPCRs were run on the CFX 96 real-time PCR cycler (Bio-Rad, Hercules, CA, USA). For PRV-DNA detection, the PerfeCTa qPCR ToughMix (Quanta BioSciences, Gaithersburg, MD, USA) was used. The temperature profile used was 3 min at 95 °C, followed by 45 cycles of 15 s at 95 °C, 15 s at 60 °C, and 15 s at 72 °C. For the RNA viruses the AgPath-ID One-Step RT-PCR Reagents of Applied Biosystems (Waltham, MA, USA) was applied. The temperature profile used here was 10 min at 45 °C, 10 min at 95 °C, followed by 45 cycles of 15 s at 95 °C, 20 s at 56 °C, and 30 s at 72 °C. Fluorescence values (FAM, HEX) were collected during the annealing step. Samples were considered positive when the quantification cycle (Cq) values were <40.

### 2.6. Virus Isolation in Cell Culture

For the virus isolation experiments of PRV, the PCR positive brain sample was used. First, 200 µL of brain material homogenized in the TissueLyser II was added to 3 mL of cell culture medium containing antibiotics (20,000 µg/mL Penicillin, 20,000 units/mL Streptomycin, 10 mg/mL Gentamicin, 250 µg/mL Amphotericin (Thermo Fisher Scientific, Waltham, MA, USA)) on one-day-old confluent PK15 (porcine kidney) cells (FLI cell culture collection number RIE0005-1); then, the T25 cell culture flask was incubated for 90 min at 37 °C. After this virus adsorption phase, the inoculum was removed, and the cells were carefully washed once with 5 mL of medium. The cell culture flask was then filled with 10 mL of medium supplemented with 10% FCS containing antibiotics and incubated for 5 days at 37 °C.

### 2.7. Whole Genome Sequencing and Assembly

Genomic DNA was extracted from the cell culture material using the NucleoMag Vet kit as described in Section 2.5 and was subsequently sent to Eurofins Genomics (Ebersberg, Germany) for sequencing using Illumina and Nanopore technology. Illumina raw reads were trimmed for quality and adapter contamination using TrimGalore (v0.6.10) and Cutadapt (v4.0; [36]) running in automatic adapter detection mode. A *de novo* assembly using the untrimmed Nanopore reads alone was done using Flye (v2.9.6-b1802; 2 iterations; [37]), and the resulting contigs were aligned to a the PRV RefSeq reference NC_006151 using Minimap2 (v2.24, map-ont preset; [38]). A draft genome was built from the mapping contigs and subsequently confirmed and corrected by mapping the Illumina reads using Bowtie2 (v2.5.4; [39]). ORFs were predicted using Geneious Prime (v2025.1.3) and annotated according to reference NC_006151. The complete and annotated genome sequence was submitted to GenBank and is available using the accession number PV962835.

### 2.8. Phylogenetic Analysis

The complete PRV genome from the examined wolf was aligned to 131 complete PRV reference sequences from GenBank using MAFFT (v7.4.9; [40]), and the redundant portion of the alignment reflecting the terminal repeat flanking the U_S_ genome region was removed. The non-redundant whole genome alignment was further cleaned using CIAlign (v1.1.4; [41]) with default parameters and used for maximum-likelihood phylogenetic reconstruction in IQ-TREE (v3.0.1; [42]) using automatic model selection [43] and 10,000 ultra-fast bootstrap replicates [44]. The resulting tree was visualized using R (v4.3.1). Furthermore, the gene UL44 encoding glycoprotein C (gC) was extracted from the complete PRV genome sequence and aligned with publicly available gC sequences from GenBank using Muscle (v3.8.425; [45]). The gC gene alignment was used for phylogenetic reconstruction as described for the whole genome alignment but with additional SH-aLRT branch support test [46]. Both alignments were visually inspected using Geneious Prime.

## 3. Results

### 3.1. Physical Examination and Necropsy

In February 2025, a ten-month-old male wolf was found moribund and showing advanced neurological symptoms in Magura National Park, in the Polish part of the Carpathian Mountains (south-eastern Poland; Figure 1). The clinical symptoms indicated severe epileptic seizures, occurring every several minutes, accompanied by vocalizations during the ictal episodes (Video S1). The following clinical signs were also observed, including lateral recumbency, marked generalized weakness, a profound reduction in spontaneous motor activity, muscle tremors, tonic–clonic spasm, irregular head movements, excessive salivation, open-mouth breathing, and tachypnea. Samples from the animal were collected postmortem several days after death.

A complete evaluation of postmortem changes was not possible due to the time elapsed between the animal’s death and the necropsy. The carcass was in moderate decomposition, with evident features of soft-tissue autolysis and internal-organ degradation. The delay in tissue sampling was due to administrative procedures. An initial preliminary necropsy was conducted by the veterinary inspection authority, during which samples were collected specifically for rabies testing. The carcass could only be released after rabies had been ruled out. Subsequently, the material was transferred for further scientific investigation. Therefore, the observed findings should be interpreted with caution, considering the potential occurrence of postmortem artifacts. In the radiographic examination, no metallic foreign bodies (bullets or bullet fragments) were detected. No signs of a possible road accident or other mechanical damage were found.

All rapid test results and rabies tests were negative.

None of the target analytes from the classes of rodenticides, carbamate and organophosphorus pesticides, coccidiostats, or mycotoxins were detected above the detection limits in the examined samples. The only compound identified was pentobarbital, which was exclusively detected in tissue samples originating from the animal that had undergone euthanasia, consistent with its known use as a euthanizing agent.

### 3.2. Molecular Screening and Virus Isolation

The wolf’s brain, lungs, spleen, liver, and heart were analyzed by various PCR assays. All tests for canine distemper virus, influenza A virus and canine herpesvirus 1 were negative. In contrast, real-time PCR for the detection of PRV genome in the brain yielded a clear positive result. The PRV glycoprotein B (gB) specific assay used showed a Cq value of 29.0, and the PRV UL19 specific assay used in parallel yielded a Cq value of 29.3. Lung tissue also produced positive results, but the detected PRV genome load was significantly lower (gB: Cq = 39.7; UL19: Cq = 35.9). No PRV genome was detected in the spleen, liver, or heart using both real-time PCR. Based on these results, four independent brain areas were tested again in the two PRV PCRs. The results confirmed the detection of the PRV genome. Cq values of 27.4 to 31.9 were found in the gB assay, and Cq values of 26.7 to 30.7 were detectable in the UL19 assay. The brain homogenate with the lowest Cq value (highest genome load) was then used directly for virus isolation in cell culture.

An initial cytopathic effect (CPE) with the formation of typical plaques was observed 2 days post infection. After 5 days, virus replication was stopped by freezing the T25 cell culture flask, and the success of virus replication was confirmed by UL19 real-time PCR (Cq = 12.2).

### 3.3. Genome Sequence and Phylogenetic Analysis

Sequencing of the extracted DNA from the inoculated cell culture resulted in a complete PRV genome. The total length of the viral genome was 143,395 bp, and it displayed the canonical PRV genome structure composed of a unique long (U_L_; 100,903 bp) region and a unique short (U_S_; 8830 bp) region, which are bordered by an internal and terminal repeat sequence (IR_S_, TR_S_; 16,831 bp). We identified and annotated 69 open reading frames.

**Figure 2:**
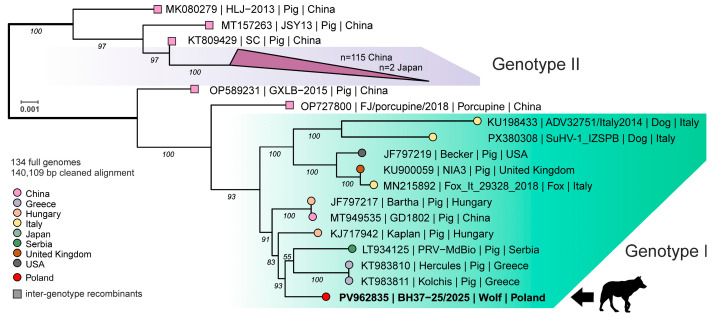
Phylogenic analysis of pseudorabies virus (PRV) based on an alignment of the whole genome from 133 reference strains along with the novel sequence from the wolf in Poland (black silhouette and arrow). The geographic origin of the strains is indicated by color, and the phylogenetic clusters that correspond to genotype I and II strains are highlighted in green and purple, respectively. Strains that did not fall within the established genotypes were considered inter-genotype recombinants (square symbols). The ultrafast bootstrap value is indicated in italics for main branches, and the scale shows substitutions per site.

**Figure 3:**
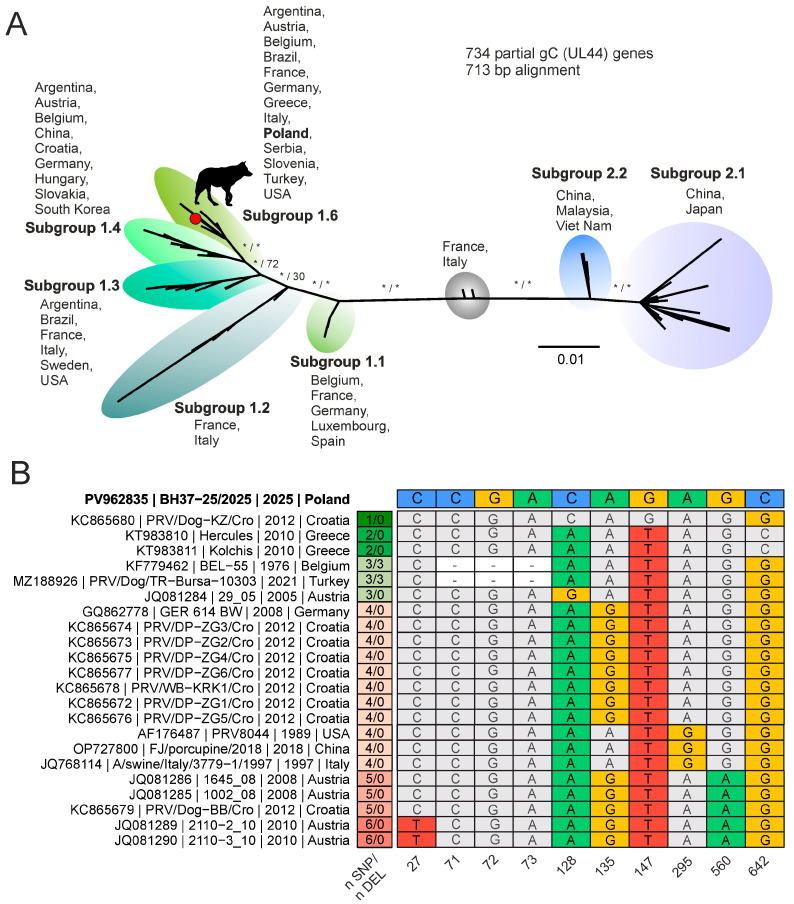
Phylogenetic analysis and nucleotide variation of the pseudorabies virus glycoprotein C (gC, UL44) gene. (**A**) Phylogenic analysis of pseudorabies virus (PRV) based on an alignment of the gC coding gene from 733 reference strains along with the novel sequence from the wolf in Poland (black silhouette). Isolates cluster into genotype I subgroups (1.1–1.6) and genotype II subgroups (2.1–2.2), highlighted by color. Statistical support is shown for main branches using the format [SH-aLRT/ultrafast bootstrap]. Asterisks indicate statistical support ≥80% and ≥95% for SH-aLRT and ultrafast bootstrap, respectively. The scale shows substitutions per site. (**B**) Alignment of selected nucleotide positions within the gC gene showing sequence variation among PRV isolates. Rows correspond to individual isolates with accession number, strain designation, year of isolation, and country of origin. The isolate from the present case (top row) is shown for comparison. Colored cells indicate nucleotide differences at specific positions within the alignment, while identical nucleotides are shown in grey. The columns on the left summarize the number of single nucleotide polymorphisms (n SNP) and deletions (n DEL) relative to the reference sequence. Positions 27, 71, 72, 73, 128, 135, 147, 295, 560, and 642 are highlighted to illustrate informative variation within and between subgroups.

Phylogenetic analysis of an alignment of 132 complete PRV genome sequences resulted in two prominent clusters consistent with previously reported genotype I and II strains. Most genotype I strains originated from Europe, while most genotype II strains originated from China. Five sequences from China did not cluster well with the established groups and were considered potential inter-genotype recombinants. The newly obtained PRV genome sequence from the Polish wolf clearly clustered with sequences of genotype I (Figure 2). Based on whole-genome alignment, PRV sequences from Serbia (PRV-MdBio; LT934125) and Greece (Kolchis; KT983811) were genetically the most closely related, with 98.6% nucleotide identity. The mean nucleotide identity of the new sequence to full PRV genomes from genotype I was 97.5% (*n* = 11) and 94.2% (*n* = 117) for genotype II. As the number of full PRV genome sequences from genotype I is limited, we also compared sequences of the gene coding for glycoprotein C (gC; UL44), which resulted in a 713 bp alignment of 734 sequences. Phylogenetic analysis of the gC alignment followed the overall separation into the two genotypes that were further separated into the six subgroups 1.1-6 as defined by Ye et al. 2015 [47]. The novel PRV sequence from the Polish wolf clustered into subgroup 1.6 along with sequences from Europe, South- and North America (Figure 3A). Based on gC, the virus that is genetically most closely related to the PRV from the Polish wolf was detected in Croatia (KC865680; PRV/Dog-KZ/Cro) and differs from it at only one site (position 642 of gC in PV962835) (Figure 3B). Two sequences from Greece differed at two sites from the new PRV sequence from Poland (positions 128 and 147 of gC in PV962835). While the differences observed at positions 147 and 642 are synonymous and do not alter the amino acid sequence, the substitution at position 128 results in a non-synonymous change in gC (A128C ↔ E43A).

## 4. Discussion

To the authors’ best knowledge, this is the first report of Aujeszky disease (AD) in a wolf in Central Europe with genomic characterization. As wild boars are important prey for wolves in Poland [26,27], and wolves also scavenge on wild boars that have died from African swine fever [48], we suspect the young wolf contracted the infection by consuming the infected meat of a killed wild boar or its carcass. Serological tests for antibodies against PRV in wild boars from the area where the wolf was found (Podkarpackie Voivodeship) showed seroprevalence ranging from 21.5 to 27.1% during the research period 2011–2014 [11]. The existence of a PRV reservoir among wild animals (mainly wild boars) has been reported in many European countries, and this situation does not affect the eradication status of the virus among domestic pigs [49,50,51,52].

The infection with PRV is fatal to wolves and other canids [53]. It is therefore one of the pathogens that can limit the wolf population (such as rabies virus, distemper, parvovirus, or sarcoptic mange). Cases of AD in wolves are unlikely to be significant in the transmission of PRV, as in most scenarios, death occurs shortly after the onset of clinical signs [53]. The symptoms of the disease in the described wolf were very similar to those described in a wolf (animal D) from Italy [22].

The detected PRV genome load in brain tissue was comparably low (Cq values 29.0 and 29.3). However, a high Cq value in brain tissue does not argue against PRV infection in general or hint towards bad sample preservation. In dogs and other carnivores, PRV is thought to spread from the site of entry via peripheral nerves, with abdominal and trigeminal ganglia commonly involved, while meningoencephalitis is often concentrated in the brainstem and spinal cord rather than being evenly distributed throughout the central nervous system [53]. Consistent with this distribution, pathological studies in dogs have shown the most prominent lesions and viral antigen in the brainstem, with only limited changes in the cerebrum and cerebellum [54]. A recent canine case report identified the brainstem as the sample with the lowest cq value among the tested tissues, while cerebellum and brain had Cq values comparable to this study [22].

Neurological symptoms in wolves with AD may resemble those of rabies. In the Podkarpacie Voivodeship (the area where the examined wolf was found), dead wolves or wolves exhibiting nervous symptoms are usually tested for the presence of the rabies virus. The same procedure was followed in this case. If the result of this test is negative, no further diagnostics are performed. It is therefore possible that AD in wolves in Poland is higher than reported. So, adding PRV testing to the routine diagnostics of wolves that are found could provide valuable information on the occurrence of the virus in the wildlife. Wolves’ diet has been intensively studied in whole Poland [26,27,28,55,56,57,58,59,60], and although juvenile wild boars are mainly preyed upon by wolves [61], sick adults are also susceptible to wolf predation, and dead wild boars are scavenged [48]. Therefore, the detection of PRV in wild boars will not vary depending on the age of the boars consumed by wolves [62]. The genomic sequence of the PRV isolate from this case matched those classified as genotype I, the most commonly detected in Europe. However, the precise geographical origin, distribution, and evolutionary history of this viral strain cannot be fully assessed, as there are very few reference sequences available. This underscores the need for more comprehensive analyses of European PRV strains and highlights the importance of full-genome sequencing in improving our understanding of viral evolution, transmission dynamics and the emergence of recombinant strains.

## Data Availability

The annotated viral genome was uploaded to GenBank using the accession PV962835 (BioProject: PRJNA1292117).

## Supplementary Materials

The following supporting information can be downloaded at https://www.mdpi.com/article/10.3390/v18040449/s1, Video S1: Aujeszky’s disease in a grey wolf (*Canis lupus*).

## Abbreviations

The following abbreviations are used in this manuscript:

AD	Aujeszky’s Disease
PRV	pseudorabies virus
gC	glycoprotein C
